# F. J. A. Mayes

**Published:** 1939

**Authors:** 


### F. J. A. MAYES, M.R.C.S., L.R.C.P.

It is with great regret that we have to record the death of Dr. F. J. A. Mayes, who died suddenly on Sunday, 3rd September, after a long period of invalidism.

Mayes was born in 1875, and after spending his school days at Bath entered as a student at University College Hospital. Immediately after qualifying he went out to the South African War. While on service there he contracted typhoid fever and was invalided home. The war experience encroached on the time that he would have spent as a resident at University College Hospital, and so shortly after his return to England he took up practice in Lancashire. This experience was to serve in after years as a rich store of anecdotes, which, given in inimitable Lancashire dialect, was a never ending source of delight and amusement to his friends.

In 1910 he married a Bristol lady, a daughter of the late Alderman Gardiner, and in 1911 he settled down in Bristol and in the course of the next few years built up an extensive practice.

In the Great War he joined the South Midland Field Ambulance and went to France with them in 1916. The rigours of service in the Field broke down a constitution not too strong, and in 1917 Mayes was invalided out.

He had, while in France, become very interested in radiology and X-ray therapy, and on his return to civil life he took up this branch of work, relinquishing general practice altogether in 1923. He was appointed radiologist at the Bristol Royal Infirmary and held that appointment until two years ago, when he retired under the age limit.

Socially, he was a delightful companion, and his ready wit and gift as a raconteur captured the affection of all who came in daily contact with him, and to these his going leaves a heartache which will not be gainsaid.

